# Noninvasive prenatal paternity determination using microhaplotypes: a pilot study

**DOI:** 10.1186/s12920-020-00806-w

**Published:** 2020-10-23

**Authors:** Jaqueline Yu Ting Wang, Martin R. Whittle, Renato David Puga, Anatoly Yambartsev, André Fujita, Helder I. Nakaya

**Affiliations:** 1 Department of Clinical Toxicological Analyzes, School of Pharmaceutical Sciences, University of São Paulo, São Paulo, Brazil; 2 Genomic Engenharia Molecular Ltda, São Paulo, Brazil; 3 Hospital Israelita Albert Einstein, São Paulo, Brazil; 4 Statistics Department, Institute of Mathematics and Statistics, University of São Paulo, São Paulo, Brazil; 5 Department of Computer Science, Institute of Mathematics and Statistics, University of São Paulo, São Paulo, Brazil

**Keywords:** Microhaplotype, Noninvasive, Probability of paternity, Prenatal, Single nucleotide polymorphism

## Abstract

**Background:**

The use of noninvasive techniques to determine paternity prenatally is increasing because it reduces the risks associated with invasive procedures. Current methods, based on SNPs, use the analysis of at least 148 markers, on average.

**Methods:**

To reduce the number of regions, we used microhaplotypes, which are chromosomal segments smaller than 200 bp containing two or more SNPs. Our method employs massively parallel sequencing and analysis of microhaplotypes as genetic markers. We tested 20 microhaplotypes and ascertained that 19 obey Hardy–Weinberg equilibrium and are independent, and data from the 1000 Genomes Project were used for population frequency and simulations.

**Results:**

We performed simulations of true and false paternity, using the 1000 Genomes Project data, to confirm if the microhaplotypes could be used as genetic markers. We observed that at least 13 microhaplotypes should be used to decrease the chances of false positives. Then, we applied the method in 31 trios, and it was able to correctly assign the fatherhood in cases where the alleged father was the real father, excluding the inconclusive results. We also cross evaluated the mother-plasma duos with the alleged fathers for false inclusions within our data, and we observed that the use of at least 15 microhaplotypes in real data also decreases the false inclusions.

**Conclusions:**

In this work, we demonstrated that microhaplotypes can be used to determine prenatal paternity by using only 15 regions and with admixtures of DNA.

## Background

Cell-free foetal DNA (cffDNA) has become an object of study for multiple purposes, including aneuploidies, monogenic disorders and paternity determination [1–3]. Commercially available tests can be performed at around 10 weeks of pregnancy and apply the use of massively parallel sequencing (MPS) [1]. By using this approach, the pregnant woman avoids invasive procedures, such as chorionic villus biopsy and amniotic fluid sampling, and their associated risks [4]. Despite that, there are some challenges related to the use of cffDNA, especially for noninvasive paternity determination. One is that at the 9th week of gestation the average foetal fraction of all circulating DNA is ~ 10% [5]. The other is that cffDNA is present in maternal plasma, which also contains maternal cell-free DNA [1].

Traditionally, short tandem repeat (STR) loci are the genetic markers used for paternity tests. However, the use of MPS to access STR loci presents two major problems: a considerable amount of stutter [6] and reduced size of maternal and foetal DNA fragments in maternal plasma [7]. The stutter hinders the allelic assignments, which is important since it is a mixture of DNA. Maternal and foetal fragments are 166 and 145 bp respectively [7], which rules out the use of several STR loci.

Single nucleotide polymorphisms (SNPs) can also be used as markers. Natera, Inc. developed a commercial noninvasive prenatal paternity test [2], which used approximately 317,000 genome-wide SNPs to enable the statistical inferences [8]. DNA Diagnostics Center (DDC) [9] assayed 2688 SNPs. Yang et al. [10] experiment described the use of semiconductor MPS to sequence SNPs. A recent study reported the use of an average of 148 effective SNPs to calculate the probability of paternity [11]. Although SNPs can be an alternative genetic marker, a large number of loci are still necessary to perform the test.

Because of the inherent difficulties in analyzing STRs in maternal plasma, we also evaluated the applicability of using the microhaplotype loci pioneered by Kidd et al. [12, 13] for this purpose. Microhaplotypes are regions of ~ 200 bp which contain two or more SNPs and comprise at least three different haplotypes [12]. Recently these authors have extended and validated the use of these markers for forensic purposes [14] including mixture analysis [15], leading to the development and application of large panels comprising > 100 microhaplotypes [16]. A panel of microhaplotypes should be well suited for performing noninvasive prenatal paternity testing by MPS. They are adequately polymorphic, do not contain repetitive motifs which hinder MPS, and can be designed to be of a suitable size for cffDNA analysis. Here we describe the implementation of microhaplotypes for carrying out noninvasive prenatal paternity testing using semiconductor MPS.

## Methods

### Microhaplotypes

Using the report of Debeljak et al. [17], we selected 20 informative regions to be studied as genetic markers (i.e. the microhaplotypes) for prenatal paternity testing; primer sequences for each microhaplotype and their genomic intervals (GRCh37/hg19) are described in Additional file 1: Table S1. The resultant amplicon sizes range from 80 to 180 bp which are suitable for cffDNA analysis and semiconductor sequencing.

Using data from the 1000 Genomes Project (1 KG, phase 3; https://www.internationalgenome.org/) [18], we also tested for and excluded allelic association for those haplotypes that are relatively close to one another on the same chromosome. We selected the microhaplotypes because of their apparent high informativeness. Besides, there is no a priori reason to believe that these segments exist in other single-copy intervals in the human genome. However, we cannot exclude this possibility at this stage. We note that Debeljak et al. [17] do not refer to these loci as ‘microhaplotypes’ and those listed in their Table 2 were derived from analyzing only three major populations, CEU, JPTCHB (combined Asian population of JPT and CHB) and YRI within 1 KG (more details about this populations is in Additional file 1: Table S2). We selected some of these loci to initiate this study knowing that not all the populations in 1 KG were covered in the initial search, and we subsequently confirmed their informativeness in all 26 1 KG populations, as we now describe.

We obtained the VCF files of 2504 samples of 26 different populations from the 1 KG. Since the genotype data are in phase, we wrote a script (available on the laboratory GitHub: https://github.com/csbl-usp/NIPT) to extract the sample’s haplotypes. We quantified these haplotypes (Additional file 1: Table S1) and calculated their frequencies (Additional file 1: Section 2, from Table S3 to Table S22), which were, subsequently, used to calculate the cumulative probability of paternity, *W* (“Probability of paternity” section). We also calculated the effective number of alleles (A_e_) for each locus and these are shown in Additional file 1: Table S23. To complete the microhaplotypes analysis, we performed HWE and LE analyses.

### Human samples

We obtained human sequencing data from 31 “trios” undergoing routine prenatal paternity testing. All the sequencing data, for each sample, was comprised in a BAM file [19]. They were generated by Genomic Engenharia Molecular Ltda and available at https://genomic.com.br/banco-de-dados/. It collected and processed samples as described by Whittle et al. [20]. Prenatal paternity testing done in parallel by DDC was used to compare our results.

### Sequencing data

To extract the data from BAM files we used the SAMtools package and our custom script [19]. All the scripts are freely available in our GitHub repository (https://github.com/csbl-usp/NIPT). The SAMtools package was used to extract the sequence data from the BAM files, and the script performed some quality filtering steps. First, we checked if the read was part of a given microhaplotype. Then the read had to be aligned to just one region, the mapping quality had to be greater than 20 (Phred scale) and the cigar string should only report matches and mismatches. With these steps, we had for each read the sequence of bases for each nucleotide position.

We extracted the SNP information, based on the position of each sequenced base. We also obtained the quality information of each base. We then substituted the bases whose quality was lower than 20 (Phred scale) with a “-”, which indicates an unknown base. Using the SNP's bases extracted from the read, we obtained a haplotype. We listed the haplotypes that had all the bases (*total haplotype*) and the haplotypes that had any “-” (*partial haplotype*). If the *partial haplotype* had less than 70% of its bases present, it was discarded and, if it had more, we tried to pair it with one *total haplotype*. The *partial haplotype* was only accepted if it matched only one *total haplotype*, otherwise, it was also discarded.

After performing the above steps for all the reads that were aligned on the analyzed region, we acquired a list of possible haplotypes (and their relative frequencies) for each microhaplotype of each sample. We used this list to determine the genotype of each microhaplotype for each mother and alleged father, only the genotyped haplotypes will be compared to the 1 KG data to obtain populational frequency. The plasma’s haplotype list was used to check the mother’s haplotypes and determine the foetal fraction and the haplotype inherited from the father.

Based on a review by Nilsen et al. [21], a SNP is considered suitable for analysis if the read coverage is > 20 × and can be considered heterozygous if the allelic imbalance varies between 20 and 80%. We referred to the work of Saba et al. [3] in which maternal plasma samples were used to diagnose a foetus with a mutation causing beta-thalassemia; here the analysis was considered reliable if the plasma DNA had an MPS coverage ≥ 1000 × per locus. We note that Saba et al. [3] used SNPs on their analysis, and since we are analysing each sequencing read, we could also use their approach. Additionally, and based on this report, we first assumed that the foetal fraction inherited from the father (half of the total foetal fraction) could vary between 1.4% and 11%.

Considering the above reports, the sequencing errors inherent to semiconductor MPS and the close similarities between different haplotypes at a given locus (as we observed empirically), we established sequential criteria to decide whether a haplotype could be identified amongst the MPS reads analyzed, as listed in Table 1. A more detailed analysis of trios sequencing data is provided in Additional file 1: Section 4 and Section 5.

**Table 1:** Sequential criteria enabling the identification of haplotypes in the mother and alleged father, based on the relative frequencies of “haplotypes” observed at a given locus

Condition 1	Condition 2	Significance
One “haplotype” > 10%	Same “haplotype” > 80%	Homozygous
Two “haplotypes” > 10%	One “haplotype” > 80%	Homozygous
Two “haplotypes” > 10%	Two “haplotypes” 20–80%	Heterozygous
Three “haplotypes” > 10%	Two “haplotypes” > 35%	Heterozygous

Provided that the maternal and paternal haplotypes can be identified and that the number of maternal plasma reads is > 1000 ×, we can then consider the evidence indicating the possibility of paternity.

### Probability of paternity

We tested the selected microhaplotypes and ascertained that 19 of the 20 selected loci obey Hardy–Weinberg equilibrium (HWE) and are independent, therefore we can calculate the paternity index (PI) of each microhaplotype and the cumulative probability of paternity (*W*). Both equations are well established in forensic analysis. To do such calculations, we need to know which haplotypes are present in the trios and their population frequencies.

We obtained the haplotypic (within each microhaplotype) frequencies using 1 KG data (see Additional file 1: Table S3 to Table S22), this is our population frequency data. We then used the mother, the alleged father and the plasma sequencing data to determine their respective haplotypes, after genotyping, we compared to the haplotypes present in 1 KG data. Using all this information, we were able to calculate the PI of each microhaplotype and then *W*.

Regarding mutations, we assume that the mutation rate of a SNP is 10^–8^ per locus per generation [22, 23]. We also consider that the lowest population frequency that a haplotype may have, based on 1 KG data, is 2 × 10^–4^. Therefore, if a mutation occurs within a haplotype, the highest PI for a locus is 5 × 10^–5^. Since this value is small, we assume that even if more than one mutation occurs within a microhaplotype, the PI will be the equivalent to one mutation. If no haplotype of the father was consistent with plasma information, we considered that a mutation has occurred.

For the haplotypes that had never been seen, we attributed their population frequency to be 1/5009. We calculated this frequency based on the 1 KG data, it has 2504 samples, summing to 5008 haplotypes.

## Results

### Paternity simulation using the 1000 Genomes Project data set

**Fig. 1:**
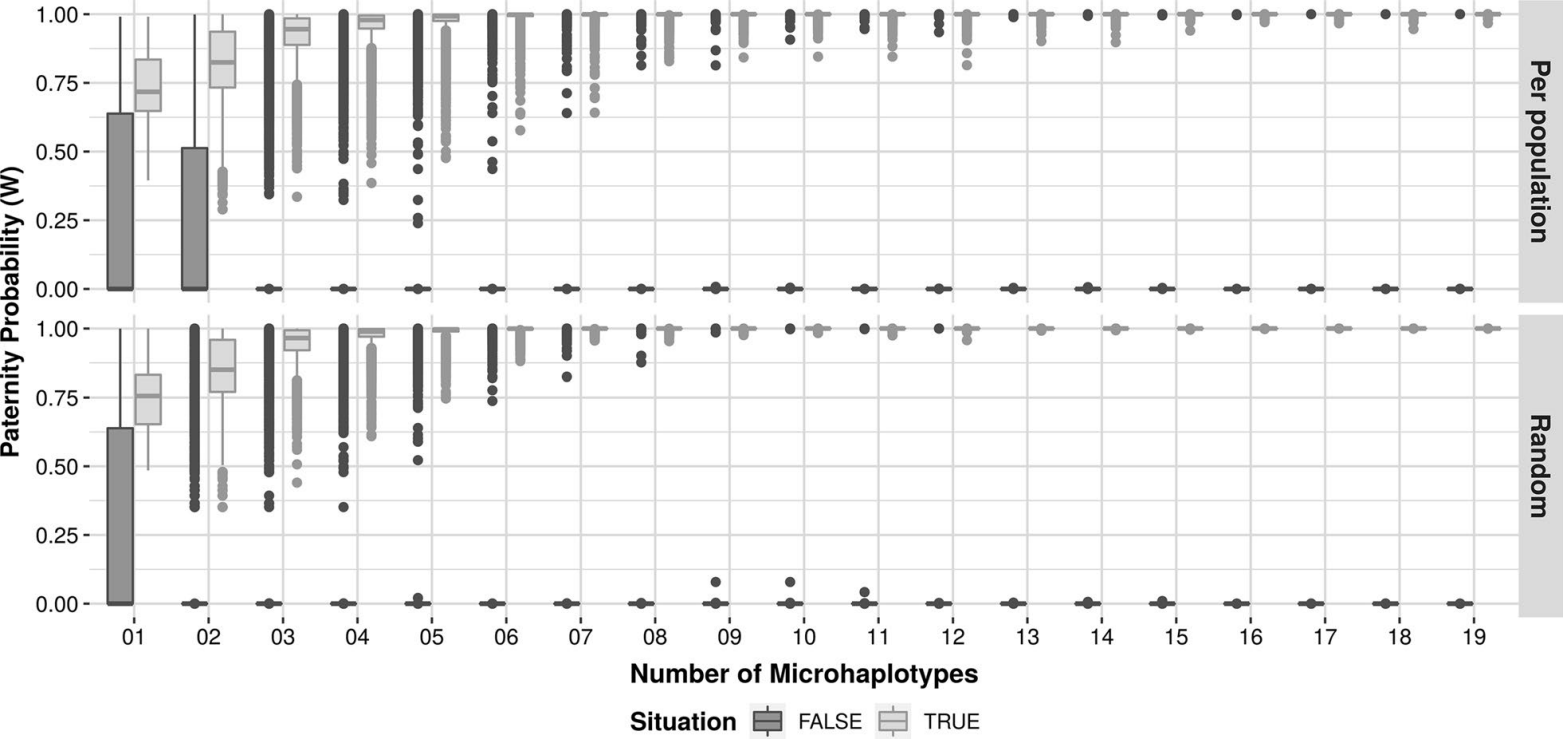
Boxplot of paternity and non-paternity simulation using 1 KG data. In dark grey, we simulated the false paternity and in light grey, we simulated the true paternity. For each situation (per population and random whole population), the simulation was repeated 26,000 times

To confirm that this set of genetic markers could be used to make a paternity analysis, we performed a simulation using 1 KG data. First, we randomly selected a man and a woman, from the same population in the 1 KG data set, and extracted their haplotypes for the 20 selected microhaplotypes. Then, we artificially created a “child” data, who inherited one microhaplotype from the man and one from the woman. We calculated the probability of paternity for this trio. We varied the number of inherited haplotypes up to 20, always calculating the probability of paternity. We repeated the simulation 1000 times for each of the 26 different populations within 1 KG and we can see the result in Fig. 1 (*Per population*, light grey boxes). To analyze the impact of both parents being selected randomly from any population, we repeated the simulation 26,000 times (to be consistent with the previous simulation) utilizing all the 1 KG data, Fig. 1 (*Random*, light grey boxes).

To simulate a “non-father” case, we repeated the previous simulations (*Random* and *Per population*) using a second man from the data whom we knew could not be the true father (Fig. 1—dark grey boxes). More details about these simulations are in Additional file 1: Section 3.

This corroborates that, as more microhaplotypes are used in the analysis, the accuracy of the calculation of the probability of paternity increases, with a decreasing possibility of false inclusions (favouring paternity). Figure 1 shows that, upon employing approximately 13 microhaplotypes, the chance of occurring a false inclusion diminishes satisfactorily (6 cases in 52,000, considering 26,000 simulations “Per population” plus 26,000 “Random”).

### Foetal fraction

According to Saba et al. [3], the appropriate foetal fraction interval to correctly identify the paternal haplotype is between 1.4 and 11%. To verify if the observed foetal fractions were inside this interval, we examined trios in which the alleged father was known to be the true father.

We analyzed the plasma sequencing data as described above and selected the haplotypes that were different from the mother but matched the alleged father. We confirmed that the foetal fraction derived only from the alleged father had a concentration within the expected interval. However, there were some exceptions, and they were considered as sequencing errors (a more detailed analysis is provided in Additional file 1: Section 5).

A foetal fraction derived solely from the alleged father and greater than 12.5% would imply a total foetal fraction of approximately 25%. This situation was not expected given the known gestational ages of the participating mothers. Therefore, we decided to discard the microhaplotypes in which it happens. We observed that the errors inherent to the sequencing technique had relative frequencies in the same range as that of the foetal paternal haplotypes. Conversely, very low foetal fractions could not be accepted. Therefore, taking these observations into account and seeking reliable information, we established that the foetal fraction (inherited from the alleged father) detection interval should be between 1 and 12%.

Foetal and maternal haplotypes should be observed in plasma sequences, and the maternal profile should predominate. We settled for a minimum cut-off of 12% for the maternal haplotypes’ relative frequencies in the plasma. Additionally, no other haplotype than the mother’s could have a relative frequency above this threshold and, if observed, the locus would be dismissed from further analysis.

**Fig. 2:**
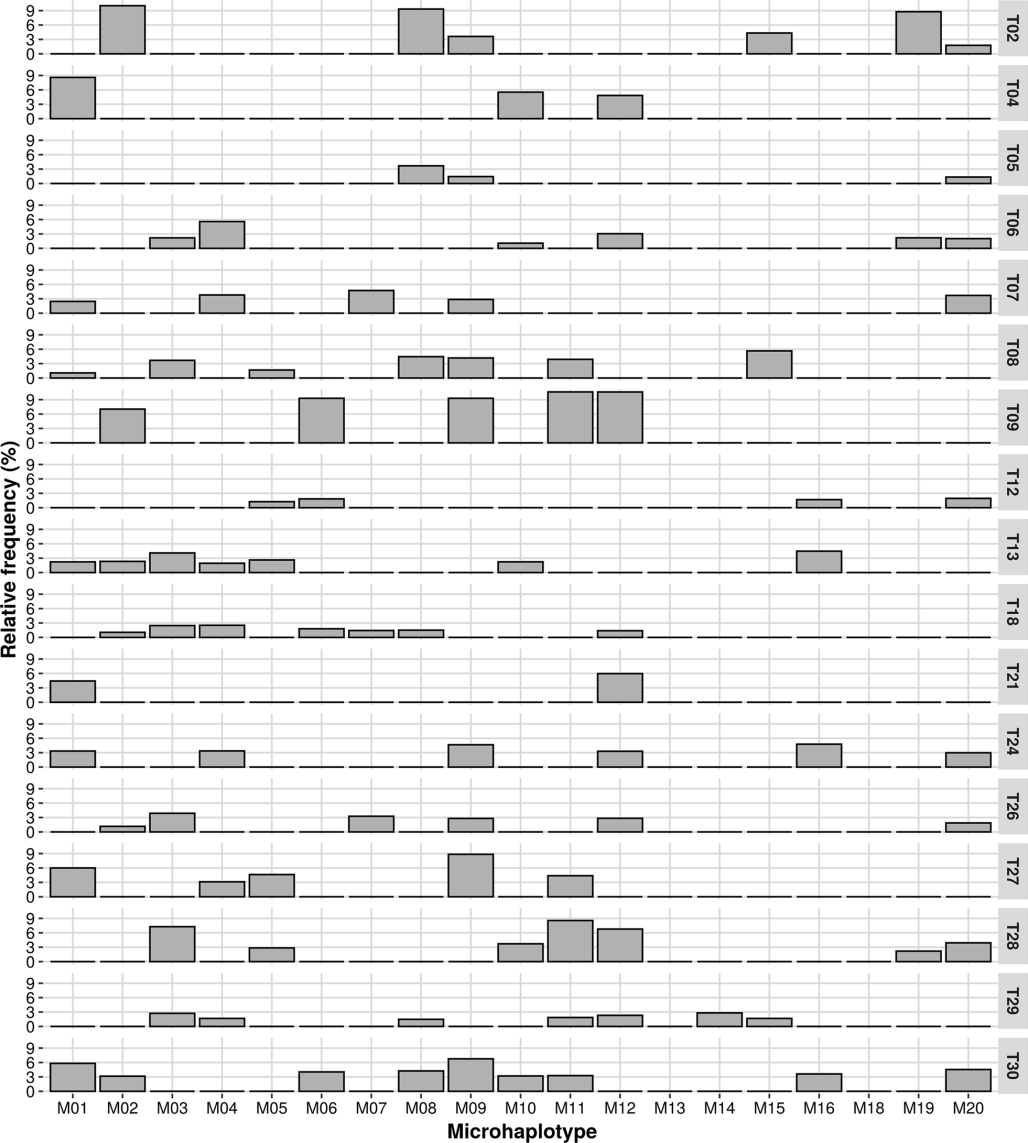
Bar plot of the relative frequency of the foetal haplotype inherited from only the father, inside the plasma’s sequencing data. Each horizontal line represents a trio for which it was known that the alleged father was the true father. The columns represent the 19 microhaplotypes used in the analyses

For each trio in which the alleged father was known to be the true father, we obtained the relative frequency of the haplotype that the foetus may have inherited from the father, as shown in Fig. 2.

As the inheritance of genetic information from the father and mother is random, we cannot define the minimum number of microhaplotypes where we should find a haplotype that the foetus inherited solely from its father. In Fig. 2, we can see that the number of observable paternal haplotypes varies between trios, for example, T04 has three microhaplotypes and T30 has nine. Although in both trios the alleged father is the true father, the number of microhaplotypes that the fetus inherited solely from its father varied in number and in combination.

Since the foetal fraction is related to the presence of foetal DNA in plasma, it was expected that the relative frequencies of observed paternal haplotypes would be proportional between the microhaplotypes of a given trio. From Fig. 2, we observe that the relative frequency of the detected paternal haplotype is not constant between the microhaplotypes, as seen in T28 and T30.

### Evidence for paternity

When we evaluated the trios’ sequencing data, we noticed that there are different combinations of observed haplotypes that may result in the possibility or not of the alleged father being the true father. We listed these combinations in Table 2.

**Table 2:** Combinations of haplotypes in alleged father, mother and plasma, and the result

*|SP*_*m*_* ∩ M*_*m*_*|*	*|FF*_*m*_*|*	Result	*|SP*_*m*_* ∩ M*_*m*_*|*	*|FF*_*m*_*|*	Result
0	0	Cannot be father	1	0	Can be father
0	1	Can be father	1	1	Can be father
0	2	Can be father	2	0	Can be father

Given a microhaplotype *m*, we analyze the observed haplotypes and determine whether or not there is evidence that the alleged father is the true father. *|SP*_*m*_* ∩ M*_*m*_*|* is the number of haplotypes in common between alleged father and mother. |*FF*_*m*_| is the number of plasma haplotypes that matched the alleged father’s haplotypes and are different from the mother’s (they are within the interval of 1% and 12%). Where *|SP*_*m*_* ∩ M*_*m*_*|*= 0 and |*FF*_*m*_|= 2 means that we found in the plasma both of alleged father’s haplotypes, and we assumed that one of them is an error and the other is the true haplotype. We considered the haplotype with the highest population frequency to be the true paternal haplotype

In paternity testing, merely one exclusionary locus is considered insufficient to result in exclusion. We, therefore, stipulated that at least two discrepant loci were required to conclude in the exclusion of paternity. When we only observed one discrepant locus, the result was considered inconclusive. Upon observing no evidence of exclusion, we calculated the probability of paternity. The analysis of the evidence for paternity of each trio is described in Additional file 1: Section 6.

### Probability of paternity

In “Paternity simulation using the 1000 Genomes Project data set” section we showed that the use of 13 microhaplotypes gave a low false-paternity inclusion rate (6 in 52,000). However, we decided to use less in our analyses because some of the 31 trios’ sequencing data had an insufficient quality and because we wished to evaluate the impact of this choice on the occurrence of false inclusions.

Considering the 2504 individuals contributing to the 1 KG data, we calculated the population frequency of each microhaplotype (Additional file 1: Section 2, Table S3 to Table S22). We used these frequencies to calculate the PI of each microhaplotype and *W*. Considering the cases where the alleged father is the real father, we correctly assigned the inclusion of paternity and the value calculated for *W* was above 99%.

**Fig. 3:**
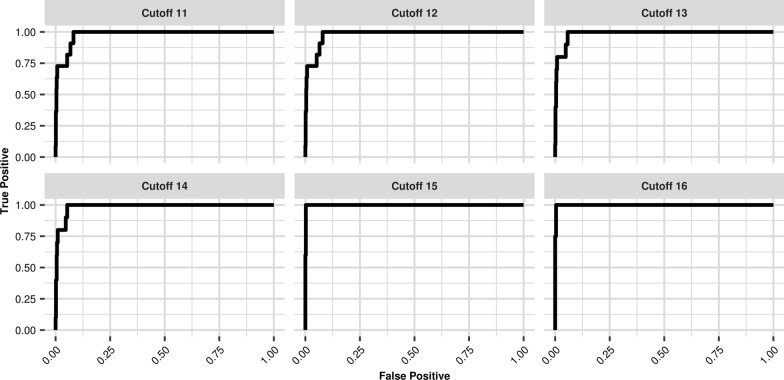
ROC curves for different cutoff numbers of microhaplotypes used. Each graph represents a minimum number of microhaplotypes used for paternity probability calculation

We also evaluated the occurrence of false paternity inclusions in our data. For this, we compared each mother-plasma duo with each alleged father. Knowing the results of each comparison, and their actual configuration, we constructed ROC curves for each minimum of microhaplotypes used in the analysis, as shown in Fig. 3. We excluded the cases where there was only one locus indicating the exclusion, because this result is inconclusive, as seen in “Evidence for paternity” section.

## Discussion

From the 1 KG data, we obtained the haplotypes from the 26 populations present in the database. We confirmed that 19 out of the 20 microhaplotypes chosen for this work were sufficiently polymorphic and obey HWE and linkage equilibrium. New informative microhaplotypes have also been described [24, 25].

By performing true and false paternity simulations with the 1 KG data, we could observe that the use of a larger number of microhaplotypes contributes to a decrease of false inclusions rate and an increase in the probability of paternity. Of the two simulations (26,000 for the whole 1 KG data and 1000 for each of the 26 populations) carried out, we obtained six false inclusions by employing 13 microhaplotypes.

Although we advocate the use of at least 15 microhaplotypes, our analyses were performed with fewer microhaplotypes because we wanted to evaluate the occurrence of false paternity inclusions. We then evaluated 31 trios to seek evidence of paternity inclusion and exclusion, the method was able to correctly assign the fatherhood, and in cases of inclusion, the value calculated for *W* was > 99%.

A major limitation of this study is that the unexpected noisy sequencing errors do not permit the assignment of a possible true father other than the tested alleged father. Increasing the read depth results in a proportional increase in this noise and is not a remedy. Consequently, the number of microhaplotypes analyzed needs to be raised.

By increasing the minimum number of microhaplotypes, the number of false inclusions decreased. This means that the use of at least 15 microhaplotypes as a threshold guarantees the quality of data and the optimization of our classifying method.

## Conclusions

In conclusion, despite limitations of quality of some samples, leading to less than 15 effective microhaplotypes and noisy sequencing errors, our method was able to correctly assign the fatherhood in the cases of true paternity. We demonstrated through the current pilot study that admixtures of DNA can be analyzed by our method, and we can test for paternity using a reduced number of genetic markers and regions. This method has the potential to be applied in clinical functions, more studies could be directed for relationship testing and, maybe, screening for organ rejection.

## Supplementary information

**Additional file 1.** Supplementary information.

## Data Availability

The datasets generated and analysed during the current study are available in the following repositories: 1000 Genomes Project, Phase 3—https://www.internationalgenome.org/. Human DNA sequencing of 31 trios—https://genomic.com.br/banco-de-dados/. Pipeline repository—https://github.com/csbl-usp/NIPT.

## Abbreviations

cffDNA: Cell-free foetal DNA
HWE: Hardy–Weinberg equilibrium
MPS: Massive parallel sequencing
STR: Short tandem repeat
SNPs: Single nucleotide polymorphisms
1KG: 1000 Genomes Project, Phase 3
PI: Paternity index
