# Supplementary material for: Noninvasive prenatal paternity determination using microhaplotypes: a pilot study
Source: BMC Med Genomics. 2020 Oct 23;13:157. doi: 10.1186/s12920-020-00806-w (PMC7584091; doi:10.1186/s12920-020-00806-w)
Supplement: Supplementary file 1 — Additional file 1. Supplementary information. [file 12920_2020_806_MOESM1_ESM.docx]

Supplementary information: Noninvasive prenatal paternity determination using microhaplotypes

# 1. Microhaplotypes

The 20 microhaplotypes were chosen based on a report of Debeljak et al [1] for prenatal paternity testing; primer sequences for each microhaplotype and their genomic intervals (GRCh37/hg19) are described in Supplementary Table 1. Including SNPs and the number of different haplotypes encountered in the 1000 Genomes Project (1KG, phase 3; <https://www.internationalgenome.org/>) database [2].

**Supplementary Table 1:** List of 20 microhaplotypes, their chromosome and amplified genomic interval in reference genome GRCh37/hg19, the SNPs that compose them, the number of distinct haplotypes observed in 1KG data and the primer sequences used. Oligonucleotide primers were designed with the aid of the Oligo 7 software [3]. The amplified genomic interval reflects the resultant amplicon size.

| **Name** | **Chromosome** | **Amplified genomic interval (GRCh37/hg19)** | **SNPs (rs)** | **Number of Haplotypes** | **Primer sequences** |
| --- | --- | --- | --- | --- | --- |
| M01 | 4 | 7447207-7447292 | 11721645, 11729625, 2277606, 11729649, 11729650, 7770503, 77178877, 58285307 | 28 | CCCTCCATGAGTGGAAGAAGG  AGTCCAGCCCTGGTGCTTC |
| M02 | 4 | 66995948-66996101 | 4342235, 4395555, 4365776, 2882483, 4339264, 4621490, 34805581 | 9 | GCCTTACAGAGAAAACACGCATG  GATCAATAAATAATCTTCTTTTATGGGTGTTTCTC |
| M03 | 5 | 178259755-178259922 | 77700663, 6894132, 86140852, 71611466, 4700814, 75686454, 71611467, 66505308, 1611468, 71611469, 6879858 | 29 | CGTGGCTGAGCTCCCCTTC  CCCAGAGGTTTTCTCTCATCCAATC |
| M04 | 6 | 31319430-31319581 | 9266064, 112974895, 9266065, 9405083, 114945721, 9266066, 35370128, 9266067 | 18 | GGCACTCTCTGATCACTGATCTC  GGGCTTGTCATATAAATGATTCCTTG |
| M05 | 8 | 3478360-3478540 | 58829796, 4875753, 4875754, 4875755, 3102099, 3110303 | 8 | TAAATTGATTTAACGCCCTGTTTTAG  CAGTCATGTTGGATTAGGGC |
| M06 | 8 | 6160289-6160440 | 4559261, 13260512, 4412392, 113523134, 112950194, 4615601, 4282592 | 6 | GCCCCTGAAACTTCTAAGAAAGC  CGAGAGGGGCAGATCACC |
| M07 | 9 | 95691384-95691561 | 13296762, 13300953, 13296126, 112492224, 7027556, 13294885, 76237981, 7027677, 7028639, 7027690, 7028645, 7027692 | 12 | GTCTTCCTCTAAATTGGTGCAAGC  TCAGTCTTTCAGTAGAGAGCACAG |
| M08 | 10 | 123095143-123095273 | 7899032, 75102425, 7913694, 7913820, 9421409, 9421410, 201989249, 7913709, 74158578, 7913828 | 17 | CACGCTTCCCCCAGATCTTC  ATGAGAGGCTTCCGTAGCTGATG |
| M09 | 10 | 133376250-133376411 | 200254070, 201475402, 112519395, 10830050, 10830051, 11018067, 11018068 | 12 | GCTGTTGGGTGGGTTCCTTC  GACTCTCTGCCTGGCGGTG |
| M10 | 11 | 5078972-5079145 | 9804487, 12806675, 11035381, 12790069, 139300303, 11035382, 12789835, 12789094, 12789111 | 13 | CCCACATTTAATTCCCCTTATTGTAAAC  GGGTATCCTAAATGGGATGCTG |
| M11 | 13 | 33553520-33553680 | 378609, 474054, 9315199, 60766807,  2149859, 570992, 76400999, 571057 | 48 | AAAAGGCATTCAAAAGTTGAGTCTG  CACCCAAAGGAATGCACGAATG |
| M12 | 13 | 99084144-99084291 | 11620100, 12868939, 79089579, 9554471, 12869473, 11616733 | 16 | TACAGTTAGCCCCCACCCAG  ATGCTCATTATGCTGATGGGCAC |
| M13 | 14 | 22736223-22736395 | 6572348, 6572349, 6572350, 6572351, 6572352, 6572353, 6572354, 191987151, 12880936 | 11 | GCGGAACTGGGGATRTATGTCTAC  TCCAGGGAGGTAACCTARACCTTC |
| M14 | 15 | 25047429-25047596 | 12914023, 12914028, 12914032, 12914037, 80258314, 28864389, 12914188, 12915332, 78817707, 146871786, 12900575, 12915886,  12914223 | 17 | CCASGTTTCAGGTCATCCCTGG  GAGCTTGGCAGGAACCACAG |
| M15 | 16 | 56576513-56576593 | 12444798, 417053, 12447596, 6499839, 9935553, 9934924, 9934927 | 32 | GTCATTAAAATCAATGCCTTTCAGC  ACACACCCCTTTCAAAACCC |
| M16 | 16 | 84540620-84540783 | 247858, 12598259, 28727369, 12598222, 12598261, 171576, 143449101 | 9 | GACTCTTTTAAAAATATACATCATGCTATTTC  CAGGACCAGCCTGGTCAG |
| M17 | 17 | 80804165-80804336 | 8069046, 8064468, 12945348, 9912486 | 13 | CTCTGCTGCCCCCTCCAC  CCAGGAAAGGAAGGGGAAGG |
| M18 | 18 | 631284-631458 | 11665412, 11662817, 11660198, 11665416, 28532598, 13381956, 11662827, 11665418, 10502288 | 16 | GGAAAGTAGCTCTTGGAATTATCTTC  CTGCCTGTCTGTTTCTCCC |
| M19 | 18 | 76597154-76597311 | 4799224, 4799225, 4799226, 4799227, 4799228, 4799229 | 7 | CGTGTACACAGACACACATGTG  GCTACGTGTCTATATGTGTTTTATGC |
| M20 | 20 | 1895548-1895704 | 66523711, 66600581, 73569345, 112203261,111980944, 6045399, 111751479, 113347998, 113961013, 114169528, 114623029, 76897346 | 41 | TGCCTCAGTGTCCCCACTTAG  CCTCTGTGTTTCCTCAAGTGTTTATC |

# 2. 1000 Genomes Project Database

The 1KG database is composed of 2504 samples from 26 different populations and 5 different super-populations. In Supplementary Table 2, we show the list of populations and super-populations from which the data were obtained.

**Supplementary Table 2:** List of super-populations and populations from where the 1KG samples were obtained and the number of samples of each population.

| **Super-population** | **Population** | **Number of samples** |
| --- | --- | --- |
| AFR | ACB (African Caribbean in Barbados) | 96 |
| AFR | ASW (African Ancestry in Southwest US) | 61 |
| AFR | ESN (Esan in Nigeria) | 99 |
| AFR | GWD (Gambian in Western Division, The Gambia) | 113 |
| AFR | LWK (Lugya in Webuye, Kenya) | 99 |
| AFR | MSL (Mende in Sierra Leoa) | 85 |
| AFR | YRI (Yoruba in Abadan, Nigeri) | 108 |
| AMR | CLM (Colombian in Mendellin, Colombia) | 94 |
| AMR | MXL (Mexican Ancestry in Los Angeles, California) | 64 |
| AMR | PEL (Peruvian in Lima, Peru) | 85 |
| AMR | PUR Puerto Rican in Puerto Rico) | 104 |
| EAS | CDX (Chinese Dan in Xishuangbanna, China) | 93 |
| EAS | CHB (Han Chinese in Beijing, China) | 103 |
| EAS | CHS (Han Chinese South) | 105 |
| EAS | JPT (Japanese in Tokyo, Japan) | 104 |
| EAS | KHV (Kinh in Ho Chi Minh City, Vietnam) | 99 |
| EUR | CEU (Utah residents with Nothern and Western European ancestry) | 99 |
| EUR | FIN (Finnish in Finland) | 99 |
| EUR | GBR (British in England and Scotland) | 91 |
| EUR | IBS (Iberian population in Spain) | 107 |
| EUR | TSI (Toscani in Italy) | 107 |
| SAS | BEB (Bangali in Bangladesh) | 86 |
| SAS | GIH (Gujarati Indian in Houston, TX) | 103 |
| SAS | ITU Indian Telugu in the UK) | 102 |
| SAS | PJL (Punjabi in Lahore, Pakistan) | 96 |
| SAS | STU (Sri Lankan Tamil in UK) | 102 |

Considering the 20 microhaplotypes selected for our study, we calculated the frequencies of the haplotypes using the super-populations and all groups from 1KG. We only show in the following tables, the frequency for all the data and for super-populations; the frequency for each population can be obtained using the scripts available on the laboratory GitHub (https://github.com/csbl-usp/NIPT) or upon request.

**Supplementary Table 3:** List of haplotypes obtained from 1KG for microhaplotype M01. Their frequencies were calculated based on all the populations and on the super-populations.

| **Haplotype** | **All groups** | **AFR** | **AMR** | **EAS** | **EUR** | **SAS** |
| --- | --- | --- | --- | --- | --- | --- |
| AAAAGGCA | 1 | 0 | 0 | 0 | 1 | 0 |
| AAAGGACG | 1 | 0 | 0 | 0 | 0 | 1 |
| AAAGGGCA | 1344 | 73 | 277 | 270 | 368 | 356 |
| AAAGGGCG | 23 | 0 | 1 | 8 | 10 | 4 |
| AAAGGGTA | 118 | 39 | 20 | 0 | 34 | 25 |
| AAGAAACA | 9 | 1 | 1 | 3 | 4 | 0 |
| AAGAAACG | 1700 | 322 | 183 | 557 | 286 | 352 |
| AAGAAGCA | 2 | 0 | 0 | 1 | 0 | 1 |
| AAGAGACA | 1 | 1 | 0 | 0 | 0 | 0 |
| AAGAGACG | 1 | 0 | 0 | 0 | 0 | 1 |
| AAGGGACG | 8 | 8 | 0 | 0 | 0 | 0 |
| AAGGGGCG | 48 | 2 | 15 | 0 | 23 | 7 |
| AGAGGGCA | 1 | 0 | 0 | 0 | 1 | 0 |
| AGGAGACA | 114 | 106 | 7 | 0 | 1 | 0 |
| AGGGGACG | 1 | 0 | 1 | 0 | 0 | 0 |
| AGGGGGCG | 1 | 1 | 0 | 0 | 0 | 0 |
| CAAGGGCA | 1 | 0 | 1 | 0 | 0 | 0 |
| CAGAAACG | 3 | 0 | 0 | 2 | 1 | 0 |
| CAGGGACG | 1 | 0 | 0 | 0 | 1 | 0 |
| CGAGGACG | 1 | 1 | 0 | 0 | 0 | 0 |
| CGAGGGCA | 14 | 12 | 1 | 0 | 1 | 0 |
| CGAGGGTA | 1 | 1 | 0 | 0 | 0 | 0 |
| CGGAAACG | 1 | 0 | 0 | 1 | 0 | 0 |
| CGGAAGCA | 9 | 9 | 0 | 0 | 0 | 0 |
| CGGGGACA | 99 | 96 | 3 | 0 | 0 | 0 |
| CGGGGACG | 620 | 206 | 81 | 143 | 132 | 58 |
| CGGGGGCA | 317 | 297 | 15 | 0 | 5 | 0 |
| CGGGGGCG | 568 | 146 | 88 | 23 | 138 | 173 |

**Supplementary Table 4:** List of haplotypes obtained from 1KG for microhaplotype M02. Their frequencies were calculated based on all the populations and on the super-populations.

| **Haplotype** | **All groups** | **AFR** | **AMR** | **EAS** | **EUR** | **SAS** |
| --- | --- | --- | --- | --- | --- | --- |
| AGCACGT | 1 | 0 | 0 | 0 | 0 | 1 |
| AGCGCGT | 1679 | 564 | 212 | 216 | 378 | 309 |
| AGTAAAC | 1 | 0 | 0 | 0 | 0 | 1 |
| GACAAGT | 1 | 0 | 0 | 1 | 0 | 0 |
| GACGCGT | 1084 | 292 | 165 | 85 | 210 | 332 |
| GATAAAC | 2138 | 414 | 314 | 665 | 416 | 329 |
| GATGAAC | 99 | 52 | 1 | 40 | 0 | 6 |
| GATGCAC | 1 | 0 | 1 | 0 | 0 | 0 |
| GATGCGT | 4 | 0 | 1 | 1 | 2 | 0 |

**Supplementary Table 5:** List of haplotypes obtained from 1KG for microhaplotype M03. Their frequencies were calculated based on all the populations and on the super-populations.

| **Haplotype** | **All groups** | **AFR** | **AMR** | **EAS** | **EUR** | **SAS** |
| --- | --- | --- | --- | --- | --- | --- |
| AAGATAACGCG | 1 | 1 | 0 | 0 | 0 | 0 |
| AAGTCAACGCG | 1 | 0 | 0 | 0 | 1 | 0 |
| AAGTCAATAAA | 3 | 1 | 1 | 0 | 1 | 0 |
| AAGTCAATAAG | 1 | 1 | 0 | 0 | 0 | 0 |
| AAGTCAATGCG | 1 | 0 | 0 | 0 | 1 | 0 |
| AAGTTAACGCG | 14 | 3 | 1 | 4 | 4 | 2 |
| AAGTTAATAAA | 1 | 1 | 0 | 0 | 0 | 0 |
| AAGTTAATAAG | 5 | 0 | 0 | 1 | 1 | 3 |
| ACGTTAGTAAG | 646 | 309 | 72 | 8 | 174 | 83 |
| AGGATAACGCG | 2 | 1 | 1 | 0 | 0 | 0 |
| AGGTCAGTAAA | 977 | 361 | 130 | 72 | 8 | 174 |
| AGGTTAGCGCG | 1 | 1 | 0 | 0 | 0 | 0 |
| AGGTTAGTAAA | 1441 | 297 | 264 | 406 | 197 | 277 |
| AGGTTAGTAAG | 261 | 43 | 23 | 169 | 7 | 19 |
| AGGTTGGTAAG | 377 | 92 | 63 | 132 | 57 | 33 |
| GAAATAACGCG | 2 | 0 | 1 | 0 | 1 | 0 |
| GAGATAACAAA | 1 | 0 | 0 | 0 | 0 | 1 |
| GAGATAACGCG | 1257 | 207 | 138 | 273 | 311 | 328 |
| GAGATAATAAA | 1 | 0 | 0 | 0 | 0 | 1 |
| GAGATAGCGCG | 1 | 0 | 0 | 0 | 0 | 1 |
| GAGATAGTAAG | 1 | 0 | 0 | 1 | 0 | 0 |
| GAGTCAATGCG | 1 | 0 | 0 | 0 | 0 | 1 |
| GAGTTAACGCG | 1 | 1 | 0 | 0 | 0 | 0 |
| GAGTTAATAAA | 2 | 2 | 0 | 0 | 0 | 0 |
| GAGTTAATAAG | 2 | 1 | 0 | 0 | 0 | 1 |
| GCGATAACGCG | 3 | 0 | 0 | 0 | 3 | 0 |
| GCGTTAATAAG | 1 | 0 | 0 | 0 | 0 | 1 |
| GGGATAACGCG | 1 | 0 | 0 | 0 | 0 | 1 |
| GGGATAGCGCG | 2 | 0 | 0 | 1 | 0 | 1 |

**Supplementary Table 6:** List of haplotypes obtained from 1KG for microhaplotype M04. Their frequencies were calculated based on all the populations and on the super-populations.

| **Haplotype** | **All groups** | **AFR** | **AMR** | **EAS** | **EUR** | **SAS** |
| --- | --- | --- | --- | --- | --- | --- |
| CAACACAT | 2 | 2 | 0 | 0 | 0 | 0 |
| CAACACTC | 25 | 23 | 1 | 0 | 1 | 0 |
| CAACACTT | 993 | 166 | 159 | 140 | 234 | 294 |
| CAACGCTT | 261 | 4 | 50 | 79 | 59 | 69 |
| CAATACAT | 238 | 14 | 19 | 97 | 64 | 44 |
| CAATACTT | 14 | 1 | 0 | 2 | 7 | 4 |
| CCACACTC | 264 | 61 | 76 | 5 | 113 | 9 |
| CCACACTT | 1 | 0 | 1 | 0 | 0 | 0 |
| TAACACTC | 20 | 1 | 2 | 17 | 0 | 0 |
| TAACACTT | 973 | 276 | 77 | 272 | 139 | 209 |
| TAATACAT | 44 | 39 | 1 | 2 | 2 | 0 |
| TAATACTC | 2 | 1 | 0 | 0 | 1 | 0 |
| TAATACTT | 614 | 174 | 87 | 219 | 83 | 51 |
| TAGCACTT | 133 | 39 | 2 | 58 | 3 | 31 |
| TAGCATTT | 366 | 109 | 46 | 5 | 132 | 74 |
| TCATACAT | 764 | 390 | 136 | 13 | 144 | 81 |
| TCATACTC | 293 | 22 | 37 | 99 | 24 | 111 |
| TCATACTT | 1 | 0 | 0 | 0 | 0 | 1 |

**Supplementary Table 7:** List of haplotypes obtained from 1KG for microhaplotype M05. Their frequencies were calculated based on all the populations and on the super-populations.

| **Haplotype** | **All groups** | **AFR** | **AMR** | **EAS** | **EUR** | **SAS** |
| --- | --- | --- | --- | --- | --- | --- |
| ACGCGT | 221 | 208 | 9 | 0 | 4 | 0 |
| GCAAGA | 1 | 0 | 0 | 1 | 0 | 0 |
| GCAAGT | 1803 | 342 | 293 | 384 | 454 | 330 |
| GCGAGT | 683 | 216 | 83 | 62 | 148 | 174 |
| GCGCAA | 1 | 0 | 0 | 1 | 0 | 0 |
| GCGCGT | 471 | 225 | 48 | 5 | 79 | 114 |
| GGAAGT | 1 | 0 | 0 | 0 | 1 | 0 |
| GGGCAA | 1827 | 331 | 261 | 555 | 320 | 360 |

**Supplementary Table 8:** List of haplotypes obtained from 1KG for microhaplotype M06. Their frequencies were calculated based on all the populations and on the super-populations.

| **Haplotype** | **All groups** | **AFR** | **AMR** | **EAS** | **EUR** | **SAS** |
| --- | --- | --- | --- | --- | --- | --- |
| CGAATAT | 24 | 23 | 1 | 0 | 0 | 0 |
| CGAGGAT | 1898 | 320 | 310 | 302 | 580 | 386 |
| CGAGGTC | 2 | 0 | 0 | 0 | 0 | 2 |
| TGAGGAT | 1 | 0 | 0 | 1 | 0 | 0 |
| TGGGGTC | 2021 | 687 | 251 | 387 | 282 | 414 |
| TTGGGTC | 1062 | 292 | 132 | 318 | 144 | 176 |

**Supplementary Table 9:** List of haplotypes obtained from 1KG for microhaplotype M07. Their frequencies were calculated based on all the populations and on the super-populations.

| **Haplotype** | **All groups** | **AFR** | **AMR** | **EAS** | **EUR** | **SAS** |
| --- | --- | --- | --- | --- | --- | --- |
| ATCAGGGGCGCG | 43 | 43 | 0 | 0 | 0 | 0 |
| ATCTAGGGCGCG | 1 | 1 | 0 | 0 | 0 | 0 |
| ATCTGAGGCGCG | 1 | 0 | 0 | 0 | 0 | 1 |
| ATCTGGGATATC | 5 | 0 | 2 | 2 | 1 | 0 |
| ATCTGGGGCACG | 1 | 0 | 0 | 0 | 0 | 1 |
| ATCTGGGGCGCG | 2790 | 519 | 370 | 630 | 587 | 684 |
| GCGTAAAATATC | 417 | 2 | 14 | 249 | 32 | 120 |
| GCGTAAAGCGCG | 3 | 0 | 0 | 1 | 0 | 2 |
| GCGTAAGATATC | 1501 | 532 | 293 | 126 | 382 | 168 |
| GCGTAAGGCGCG | 12 | 3 | 3 | 0 | 4 | 2 |
| GCGTGAGATATC | 3 | 3 | 0 | 0 | 0 | 0 |
| GTGTAGGACATC | 231 | 219 | 12 | 0 | 0 | 0 |

**Supplementary Table 10:** List of haplotypes obtained from 1KG for microhaplotype M08. Their frequencies were calculated based on all the populations and on the super-populations.

| **Haplotype** | **All groups** | **AFR** | **AMR** | **EAS** | **EUR** | **SAS** |
| --- | --- | --- | --- | --- | --- | --- |
| CGACGGTATA | 1 | 0 | 0 | 1 | 0 | 0 |
| CGACGGTATG | 1296 | 189 | 247 | 123 | 374 | 363 |
| CGGAAACATG | 1260 | 213 | 217 | 354 | 244 | 232 |
| CGGAAACGTG | 165 | 159 | 6 | 0 | 0 | 0 |
| CGGAGGTATG | 6 | 0 | 0 | 6 | 0 | 0 |
| TAGCAACATG | 1 | 0 | 0 | 1 | 0 | 0 |
| TAGCGGTATG | 103 | 2 | 1 | 53 | 1 | 46 |
| TGACAACATA | 2 | 0 | 1 | 1 | 0 | 0 |
| TGACAACATG | 11 | 0 | 2 | 0 | 9 | 0 |
| TGACGGTACA | 42 | 40 | 2 | 0 | 0 | 0 |
| TGACGGTACG | 230 | 209 | 16 | 1 | 2 | 2 |
| TGACGGTATA | 1177 | 137 | 134 | 376 | 292 | 238 |
| TGACGGTATG | 140 | 27 | 5 | 38 | 2 | 68 |
| TGGAAATATG | 48 | 45 | 3 | 0 | 0 | 0 |
| TGGAGGTATG | 1 | 0 | 0 | 1 | 0 | 0 |
| TGGCAATATG | 524 | 301 | 60 | 52 | 82 | 29 |
| TGGCGGTATG | 1 | 0 | 0 | 1 | 0 | 0 |

**Supplementary Table 11:** List of haplotypes obtained from 1KG for microhaplotype M09. Their frequencies were calculated based on all the populations and on the super-populations.

| **Haplotype** | **All groups** | **AFR** | **AMR** | **EAS** | **EUR** | **SAS** |
| --- | --- | --- | --- | --- | --- | --- |
| GCCGCAT | 8 | 5 | 1 | 0 | 2 | 0 |
| GCCGCGC | 749 | 406 | 90 | 102 | 72 | 79 |
| GCCGCGT | 7 | 0 | 2 | 0 | 4 | 1 |
| GCCGTGC | 1006 | 196 | 150 | 229 | 127 | 304 |
| GCCTCGC | 6 | 4 | 0 | 1 | 0 | 1 |
| GCCTTGC | 1261 | 198 | 185 | 220 | 410 | 248 |
| GCTGCAT | 15 | 6 | 1 | 4 | 2 | 2 |
| GGTGCAT | 7 | 1 | 1 | 2 | 3 | 0 |
| GGTGCGC | 2 | 0 | 0 | 0 | 2 | 0 |
| TGTGCAT | 1610 | 336 | 224 | 450 | 277 | 323 |
| TGTGCGC | 252 | 168 | 27 | 0 | 44 | 13 |
| TGTGCGT | 85 | 2 | 13 | 0 | 63 | 7 |

**Supplementary Table 12:** List of haplotypes obtained from 1KG for microhaplotype M10. Their frequencies were calculated based on all the populations and on the super-populations.

| **Haplotype** | **All groups** | **AFR** | **AMR** | **EAS** | **EUR** | **SAS** |
| --- | --- | --- | --- | --- | --- | --- |
| ACCCCGACC | 5 | 0 | 1 | 2 | 1 | 1 |
| ACCCCGGCC | 10 | 1 | 0 | 3 | 2 | 4 |
| ACCCCGGCT | 10 | 2 | 3 | 2 | 3 | 0 |
| ACCCTGGCT | 1 | 1 | 0 | 0 | 0 | 0 |
| AGTCCTACC | 1 | 0 | 0 | 0 | 0 | 1 |
| AGTTCGGCT | 4 | 1 | 0 | 0 | 1 | 2 |
| AGTTCTACC | 2517 | 522 | 340 | 737 | 488 | 230 |
| GCCCCGGCT | 2 | 2 | 0 | 0 | 0 | 0 |
| GCCCCGGGT | 2364 | 712 | 341 | 264 | 509 | 538 |
| GCCCTGGGT | 88 | 79 | 9 | 0 | 0 | 0 |
| GGTTCGGGT | 1 | 0 | 0 | 0 | 1 | 0 |
| GGTTCTGCT | 1 | 0 | 0 | 0 | 0 | 1 |
| GGTTCTGGT | 4 | 2 | 0 | 0 | 1 | 1 |

**Supplementary Table 13:** List of haplotypes obtained from 1KG for microhaplotype M11. Their frequencies were calculated based on all the populations and on the super-populations.

| **Haplotype** | **All groups** | **AFR** | **AMR** | **EAS** | **EUR** | **SAS** |
| --- | --- | --- | --- | --- | --- | --- |
| ACAACACA | 2 | 2 | 0 | 0 | 0 | 0 |
| ACAACACG | 3 | 0 | 0 | 3 | 0 | 0 |
| ACAATACA | 9 | 1 | 1 | 0 | 7 | 0 |
| ACAATGCA | 1 | 0 | 0 | 0 | 1 | 0 |
| ACAATGCG | 76 | 1 | 11 | 1 | 42 | 21 |
| ACGACACA | 4 | 0 | 0 | 1 | 3 | 0 |
| ACGACACG | 7 | 0 | 1 | 0 | 5 | 1 |
| ACGATACA | 40 | 0 | 5 | 4 | 12 | 19 |
| ACGATACG | 1 | 0 | 0 | 0 | 0 | 1 |
| ACGGCAAA | 4 | 4 | 0 | 0 | 0 | 0 |
| ACGGTGCA | 2 | 2 | 0 | 0 | 0 | 0 |
| ATAACAAA | 2 | 2 | 0 | 0 | 0 | 0 |
| ATAACACA | 152 | 64 | 19 | 35 | 27 | 7 |
| ATAACACG | 1418 | 376 | 282 | 310 | 216 | 234 |
| ATAACGCA | 1 | 1 | 0 | 0 | 0 | 0 |
| ATAACGCG | 20 | 1 | 6 | 12 | 0 | 1 |
| ATAATACA | 1242 | 266 | 166 | 234 | 231 | 345 |
| ATAATACG | 5 | 0 | 1 | 0 | 2 | 2 |
| ATAATGCA | 146 | 137 | 3 | 1 | 2 | 3 |
| ATAATGCG | 507 | 50 | 70 | 54 | 155 | 178 |
| ATAGTACA | 1 | 1 | 0 | 0 | 0 | 0 |
| ATAGTGCA | 9 | 9 | 0 | 0 | 0 | 0 |
| ATGACACA | 2 | 0 | 0 | 1 | 1 | 0 |
| ATGACACG | 29 | 2 | 2 | 12 | 4 | 9 |
| ATGATACA | 9 | 2 | 0 | 5 | 0 | 2 |
| ATGATGCG | 2 | 0 | 0 | 0 | 2 | 0 |
| ATGGCACA | 1 | 1 | 0 | 0 | 0 | 0 |
| ATGGCACG | 10 | 10 | 0 | 0 | 0 | 0 |
| ATGGTGCA | 1 | 1 | 0 | 0 | 0 | 0 |
| CCAACAAA | 94 | 88 | 5 | 0 | 1 | 0 |
| CCAACACA | 8 | 4 | 1 | 1 | 2 | 0 |
| CCAACACG | 13 | 6 | 0 | 7 | 0 | 0 |
| CCAATACA | 25 | 15 | 1 | 0 | 8 | 1 |
| CCAATGCA | 21 | 21 | 0 | 0 | 0 | 0 |
| CCAATGCG | 7 | 0 | 3 | 0 | 3 | 1 |
| CCAGTGCA | 8 | 8 | 0 | 0 | 0 | 0 |
| CCGACACA | 809 | 75 | 105 | 264 | 222 | 143 |
| CCGACACG | 55 | 3 | 7 | 17 | 23 | 5 |
| CCGATACA | 38 | 25 | 0 | 6 | 6 | 1 |
| CCGATGCG | 69 | 2 | 0 | 40 | 24 | 3 |
| CCGGCAAA | 39 | 38 | 1 | 0 | 0 | 0 |
| CCGGCACA | 57 | 56 | 1 | 0 | 0 | 0 |
| CCGGTGCA | 5 | 5 | 0 | 0 | 0 | 0 |
| CTAACACA | 1 | 1 | 0 | 0 | 0 | 0 |
| CTAACACG | 37 | 29 | 3 | 0 | 5 | 0 |
| CTAATACA | 3 | 0 | 0 | 0 | 2 | 1 |
| CTAATGCA | 12 | 12 | 0 | 0 | 0 | 0 |
| CTAATGCG | 1 | 1 | 0 | 0 | 0 | 0 |

**Supplementary Table 14:** List of haplotypes obtained from 1KG for microhaplotype M12. Their frequencies were calculated based on all the populations and on the super-populations.

| **Haplotype** | **All groups** | **AFR** | **AMR** | **EAS** | **EUR** | **SAS** |
| --- | --- | --- | --- | --- | --- | --- |
| CATCAA | 1 | 1 | 0 | 0 | 0 | 0 |
| CATCGG | 1513 | 297 | 222 | 324 | 385 | 285 |
| CATGGA | 2 | 0 | 0 | 1 | 0 | 1 |
| CGTCGG | 6 | 1 | 4 | 0 | 1 | 0 |
| TAGGGA | 2 | 1 | 0 | 0 | 0 | 1 |
| TATCGA | 115 | 26 | 6 | 31 | 4 | 48 |
| TATCGG | 358 | 104 | 67 | 143 | 4 | 40 |
| TATGAA | 1 | 1 | 0 | 0 | 0 | 0 |
| TATGGA | 1866 | 380 | 300 | 364 | 408 | 414 |
| TATGGG | 4 | 0 | 0 | 0 | 4 | 0 |
| TGGCAA | 409 | 212 | 29 | 14 | 67 | 87 |
| TGGCGA | 3 | 2 | 1 | 0 | 0 | 0 |
| TGTCAA | 721 | 291 | 65 | 130 | 133 | 102 |
| TGTCGA | 4 | 4 | 0 | 0 | 0 | 0 |
| TGTCGG | 1 | 0 | 0 | 1 | 0 | 0 |
| TGTGGA | 2 | 2 | 0 | 0 | 0 | 0 |

**Supplementary Table 15:** List of haplotypes obtained from 1KG for microhaplotype M13. Their frequencies were calculated based on all the populations and on the super-populations.

| **Haplotype** | **All groups** | **AFR** | **AMR** | **EAS** | **EUR** | **SAS** |
| --- | --- | --- | --- | --- | --- | --- |
| ACGCCACGC | 2 | 0 | 0 | 0 | 0 | 2 |
| ACGTCACGC | 22 | 0 | 1 | 3 | 0 | 18 |
| ACGTCACGT | 1098 | 301 | 175 | 117 | 300 | 205 |
| ACGTTGTGC | 3 | 0 | 1 | 0 | 1 | 1 |
| ACTCCACAC | 4 | 3 | 1 | 0 | 0 | 0 |
| ACTCCACGC | 2447 | 490 | 339 | 520 | 573 | 525 |
| ACTCTGTGC | 1 | 0 | 1 | 0 | 0 | 0 |
| ACTTTGTGC | 1 | 0 | 0 | 0 | 1 | 0 |
| ATGTTGTGC | 17 | 0 | 0 | 14 | 2 | 1 |
| GCTCCACGC | 1 | 1 | 0 | 0 | 0 | 0 |
| GTGTTGTGC | 1412 | 527 | 176 | 354 | 129 | 226 |

**Supplementary Table 16:** List of haplotypes obtained from 1KG for microhaplotype M14. Their frequencies were calculated based on all the populations and on the super-populations.

| **Haplotype** | **All groups** | **AFR** | **AMR** | **EAS** | **EUR** | **SAS** |
| --- | --- | --- | --- | --- | --- | --- |
| GGGGCGGCAATAG | 108 | 101 | 6 | 0 | 1 | 0 |
| GGGGCTACCATAG | 245 | 31 | 41 | 1 | 142 | 30 |
| GGGGCTATAACAA | 1 | 0 | 1 | 0 | 0 | 0 |
| GGGGCTGCAACAG | 2 | 1 | 0 | 1 | 0 | 0 |
| GGGGCTGCAATAA | 1 | 0 | 0 | 1 | 0 | 0 |
| GGGGCTGCAATAG | 2593 | 411 | 367 | 720 | 591 | 504 |
| GGGGCTGCCATAG | 1 | 0 | 0 | 0 | 0 | 1 |
| GGTGCTGCAATAG | 2 | 2 | 0 | 0 | 0 | 0 |
| TAGACTATAACAA | 2 | 2 | 0 | 0 | 0 | 0 |
| TATACTACAACAA | 1 | 1 | 0 | 0 | 0 | 0 |
| TATACTATAACAA | 1433 | 689 | 210 | 96 | 197 | 241 |
| TATACTATAACCA | 453 | 16 | 63 | 106 | 71 | 197 |
| TATACTATACCAA | 63 | 59 | 4 | 0 | 0 | 0 |
| TATACTGTAACAA | 2 | 2 | 0 | 0 | 0 | 0 |
| TATAGTATAACAA | 93 | 0 | 2 | 83 | 3 | 5 |
| TATGCTATAACAA | 7 | 7 | 0 | 0 | 0 | 0 |
| TATGCTATAACCA | 1 | 0 | 0 | 0 | 1 | 0 |

**Supplementary Table 17:** List of haplotypes obtained from 1KG for microhaplotype M15. Their frequencies were calculated based on all the populations and on the super-populations.

| **Haplotype** | **All groups** | **AFR** | **AMR** | **EAS** | **EUR** | **SAS** |
| --- | --- | --- | --- | --- | --- | --- |
| ACCCCGA | 2 | 0 | 0 | 2 | 0 | 0 |
| ACCCCGG | 610 | 14 | 69 | 287 | 143 | 97 |
| ACCCGCA | 487 | 185 | 68 | 182 | 3 | 49 |
| ACCCGCG | 5 | 0 | 1 | 2 | 2 | 0 |
| ACCTCGG | 26 | 22 | 1 | 2 | 0 | 1 |
| ACCTGCA | 20 | 11 | 1 | 0 | 1 | 7 |
| ACTCCCA | 5 | 3 | 0 | 2 | 0 | 0 |
| ACTCCGG | 2 | 0 | 1 | 0 | 0 | 1 |
| ACTCGCA | 1320 | 405 | 186 | 108 | 442 | 179 |
| ACTTCGG | 3 | 0 | 1 | 0 | 0 | 2 |
| ATCCCGG | 512 | 84 | 79 | 129 | 77 | 143 |
| ATCCGCA | 2 | 1 | 0 | 0 | 1 | 0 |
| ATCTCGA | 1 | 1 | 0 | 0 | 0 | 0 |
| ATCTCGG | 440 | 165 | 29 | 69 | 37 | 140 |
| ATCTGCA | 136 | 16 | 24 | 2 | 41 | 53 |
| ATCTGCG | 1 | 0 | 1 | 0 | 0 | 0 |
| ATTCGCA | 10 | 0 | 0 | 10 | 0 | 0 |
| GCCCCGG | 3 | 0 | 1 | 0 | 2 | 0 |
| GCCCGCA | 75 | 0 | 8 | 0 | 54 | 13 |
| GCCTCGG | 2 | 2 | 0 | 0 | 0 | 0 |
| GCCTGCA | 36 | 22 | 4 | 2 | 3 | 5 |
| GCTCCCA | 1 | 1 | 0 | 0 | 0 | 0 |
| GCTCCGA | 90 | 86 | 4 | 0 | 0 | 0 |
| GCTCCGG | 1 | 0 | 0 | 0 | 1 | 0 |
| GCTCGCA | 916 | 273 | 132 | 58 | 188 | 265 |
| GCTTCGG | 7 | 7 | 0 | 0 | 0 | 0 |
| GCTTGCA | 57 | 1 | 29 | 27 | 0 | 0 |
| GTCCCGG | 3 | 0 | 1 | 2 | 0 | 0 |
| GTCCGCA | 1 | 0 | 0 | 1 | 0 | 0 |
| GTCTCGG | 19 | 10 | 1 | 0 | 6 | 2 |
| GTCTGCA | 214 | 13 | 53 | 122 | 5 | 21 |
| GTTCGCA | 1 | 0 | 0 | 1 | 0 | 0 |

**Supplementary Table 18:** List of haplotypes obtained from 1KG for microhaplotype M16. Their frequencies were calculated based on all the populations and on the super-populations.

| **Haplotype** | **All groups** | **AFR** | **AMR** | **EAS** | **EUR** | **SAS** |
| --- | --- | --- | --- | --- | --- | --- |
| AGGCGCG | 836 | 279 | 126 | 270 | 36 | 125 |
| AGGCGTG | 2 | 2 | 0 | 0 | 0 | 0 |
| CCATTCG | 1568 | 390 | 178 | 192 | 496 | 312 |
| CCGCGCG | 1 | 0 | 0 | 1 | 0 | 0 |
| CCGTTCG | 1 | 0 | 0 | 1 | 0 | 0 |
| CGGCGCC | 137 | 3 | 24 | 45 | 14 | 51 |
| CGGCGCG | 1973 | 513 | 281 | 499 | 258 | 422 |
| CGGCGTC | 8 | 1 | 1 | 0 | 5 | 1 |
| CGGCGTG | 482 | 134 | 84 | 0 | 197 | 67 |

**Supplementary Table 19:** List of haplotypes obtained from 1KG for microhaplotype M17. Their frequencies were calculated based on all the populations and on the super-populations.

| **Haplotype** | **All groups** | **AFR** | **AMR** | **EAS** | **EUR** | **SAS** |
| --- | --- | --- | --- | --- | --- | --- |
| CCAC | 268 | 123 | 18 | 29 | 55 | 43 |
| CCAT | 2 | 1 | 0 | 0 | 1 | 0 |
| CCGC | 189 | 128 | 13 | 2 | 32 | 14 |
| CCGT | 1565 | 305 | 316 | 408 | 320 | 216 |
| CTAC | 2 | 1 | 0 | 0 | 0 | 1 |
| CTGC | 570 | 48 | 50 | 68 | 109 | 295 |
| CTGT | 12 | 11 | 0 | 0 | 1 | 0 |
| TCAC | 1351 | 216 | 204 | 306 | 368 | 257 |
| TCGC | 250 | 216 | 16 | 2 | 8 | 8 |
| TCGT | 523 | 58 | 65 | 188 | 88 | 124 |
| TTAC | 6 | 2 | 0 | 3 | 0 | 1 |
| TTGC | 233 | 183 | 11 | 1 | 21 | 17 |
| TTGT | 37 | 30 | 1 | 1 | 3 | 2 |

**Supplementary Table 20**: List of haplotypes obtained from 1KG for microhaplotype M18. Their frequencies were calculated based on all the populations and on the super-populations.

| **Haplotype** | **All groups** | **AFR** | **AMR** | **EAS** | **EUR** | **SAS** |
| --- | --- | --- | --- | --- | --- | --- |
| CATCATCTG | 3 | 3 | 0 | 0 | 0 | 0 |
| CATCCTCCG | 1 | 1 | 0 | 0 | 0 | 0 |
| CATCCTCTG | 3 | 3 | 0 | 0 | 0 | 0 |
| CGACACGCA | 1 | 0 | 1 | 0 | 0 | 0 |
| CGACACGCG | 1 | 1 | 0 | 0 | 0 | 0 |
| CGACATCCA | 1 | 0 | 0 | 0 | 0 | 1 |
| CGACCCCTG | 44 | 41 | 3 | 0 | 0 | 0 |
| CGACCCGCG | 2126 | 541 | 396 | 213 | 598 | 378 |
| CGACCTGCG | 80 | 77 | 2 | 0 | 1 | 0 |
| CGACTCGCG | 2 | 0 | 2 | 0 | 0 | 0 |
| TATCACCCA | 1 | 0 | 1 | 0 | 0 | 0 |
| TATCATCCA | 1969 | 551 | 247 | 465 | 313 | 393 |
| TATCATCCG | 7 | 2 | 0 | 5 | 0 | 0 |
| TATCCTCCG | 1 | 1 | 0 | 0 | 0 | 0 |
| TATTATCTG | 694 | 32 | 37 | 325 | 94 | 206 |
| TGTCATCCA | 74 | 69 | 5 | 0 | 0 | 0 |

**Supplementary Table 21:** List of haplotypes obtained from 1KG for microhaplotype M19. Their frequencies were calculated based on all the populations and on the super-populations.

| **Haplotype** | **All groups** | **AFR** | **AMR** | **EAS** | **EUR** | **SAS** |
| --- | --- | --- | --- | --- | --- | --- |
| AGTAAT | 3 | 1 | 1 | 0 | 1 | 0 |
| AGTTAT | 88 | 79 | 5 | 0 | 4 | 0 |
| AGTTGC | 2775 | 300 | 389 | 766 | 625 | 695 |
| AGTTGT | 130 | 117 | 9 | 3 | 1 | 0 |
| CACAAC | 2 | 0 | 1 | 0 | 0 | 1 |
| CACAAT | 2009 | 825 | 289 | 239 | 375 | 281 |
| CACAGC | 1 | 0 | 0 | 0 | 0 | 1 |

**Supplementary Table 22:** List of haplotypes obtained from 1KG for microhaplotype M20. Their frequencies were calculated based on all the populations and on the super-populations.

| **Haplotype** | **All groups** | **AFR** | **AMR** | **EAS** | **EUR** | **SAS** |
| --- | --- | --- | --- | --- | --- | --- |
| CCAACCCCTACT | 2 | 0 | 0 | 0 | 2 | 0 |
| CCAACCCCTCAC | 5 | 1 | 1 | 0 | 0 | 3 |
| CCGACCCCTCAC | 1 | 0 | 0 | 0 | 0 | 1 |
| CCGACCCTAACT | 1 | 1 | 0 | 0 | 0 | 0 |
| CCGCCCCCTCAC | 4 | 0 | 0 | 1 | 0 | 3 |
| CCGCTCCCTCAC | 5 | 1 | 1 | 0 | 0 | 3 |
| CCGCTCTCTCAC | 1 | 0 | 0 | 0 | 0 | 1 |
| CCGCTCTTAACT | 2567 | 707 | 367 | 349 | 626 | 518 |
| CCGCTGTTAACT | 17 | 16 | 1 | 0 | 0 | 0 |
| CTAACCCCTACT | 1 | 1 | 0 | 0 | 0 | 0 |
| CTAACCCCTCAC | 37 | 5 | 5 | 12 | 6 | 9 |
| CTAACCTTAACT | 4 | 1 | 1 | 2 | 0 | 0 |
| CTGCCCCCTCAC | 5 | 0 | 0 | 3 | 1 | 1 |
| CTGCTCCCTCAC | 5 | 0 | 1 | 0 | 3 | 1 |
| CTGCTCTTAACT | 27 | 3 | 5 | 10 | 8 | 1 |
| TCAACCCCTCAC | 1 | 1 | 0 | 0 | 0 | 0 |
| TCGACCCCTCAC | 1 | 0 | 0 | 0 | 0 | 1 |
| TCGCTCCCTCAC | 2 | 2 | 0 | 0 | 0 | 0 |
| TCGCTCTCTCAC | 1 | 0 | 0 | 0 | 0 | 1 |
| TCGCTCTTAACT | 14 | 6 | 0 | 0 | 1 | 7 |
| TCGCTCTTTCAC | 1 | 0 | 0 | 0 | 0 | 1 |
| TTAACCCCTACC | 2 | 0 | 1 | 0 | 0 | 1 |
| TTAACCCCTACT | 25 | 4 | 5 | 7 | 6 | 3 |
| TTAACCCCTCAC | 1780 | 311 | 265 | 565 | 273 | 366 |
| TTAACCCCTCAT | 1 | 0 | 0 | 1 | 0 | 0 |
| TTAACCCTAACC | 2 | 0 | 0 | 1 | 0 | 1 |
| TTAACCCTAACT | 19 | 7 | 1 | 3 | 4 | 4 |
| TTAACCTTAACC | 1 | 0 | 1 | 0 | 0 | 0 |
| TTAACCTTAACT | 63 | 18 | 6 | 17 | 12 | 10 |
| TTAATCTTAACT | 11 | 2 | 2 | 1 | 6 | 0 |
| TTAATCTTACAC | 2 | 1 | 0 | 0 | 1 | 0 |
| TTACCCCCTCAC | 1 | 0 | 0 | 0 | 0 | 1 |
| TTACTCCCTCAC | 222 | 200 | 9 | 0 | 2 | 11 |
| TTACTCTTAACT | 2 | 0 | 0 | 1 | 1 | 0 |
| TTGACCCCTACT | 2 | 0 | 0 | 0 | 2 | 0 |
| TTGACCCCTCAC | 7 | 0 | 0 | 2 | 5 | 0 |
| TTGCCCCCTCAC | 3 | 0 | 1 | 2 | 0 | 0 |
| TTGCTCCCTCAC | 40 | 4 | 3 | 8 | 10 | 15 |
| TTGCTCTCTCAC | 9 | 2 | 1 | 2 | 3 | 1 |
| TTGCTCTTAACT | 108 | 25 | 17 | 18 | 34 | 14 |
| TTGCTCTTACAC | 6 | 3 | 0 | 3 | 0 | 0 |

**Supplementary Table 23**: Allele Effective number (A_e_) calculated for each microhaplotype, using all populations and the five super-populations. Number of different alleles (N) for each microhaplotype, considering all populations and the five super-populations.

| **Microha-plotype** | **All groups (A_e_)** | **All groups (N)** | **AFR (A_e_)** | **AFR (N)** | **AMR (A_e_)** | **AMR (N)** | **EAS (A_e_)** | **EAS (N)** | **EUR (A_e_)** | **EUR (N)** | **SAS (A_e_)** | **SAS (N)** |
| --- | --- | --- | --- | --- | --- | --- | --- | --- | --- | --- | --- | --- |
| M01 | 4,52 | 28 | 6,17 | 17 | 3,84 | 14 | 2,51 | 9 | 3,96 | 15 | 3,36 | 10 |
| M02 | 2,92 | 9 | 3,03 | 4 | 2,82 | 6 | 2,04 | 6 | 2,81 | 4 | 3,05 | 6 |
| M03 | 4,79 | 29 | 4,76 | 16 | 4,18 | 10 | 3,56 | 10 | 4,39 | 13 | 3,93 | 16 |
| M04 | 7,53 | 18 | 5,71 | 16 | 6,94 | 14 | 5,95 | 13 | 7,22 | 14 | 5,80 | 12 |
| M05 | 3,42 | 8 | 4,76 | 5 | 2,95 | 5 | 2,21 | 6 | 3,01 | 6 | 3,39 | 4 |
| M06 | 2,85 | 6 | 2,65 | 4 | 2,73 | 4 | 2,97 | 4 | 2,32 | 3 | 2,72 | 4 |
| M07 | 2,44 | 12 | 2,90 | 8 | 2,16 | 6 | 2,14 | 5 | 2,06 | 5 | 1,87 | 7 |
| M08 | 4,97 | 17 | 6,63 | 10 | 3,71 | 12 | 3,52 | 13 | 3,47 | 8 | 3,83 | 7 |
| M09 | 4,30 | 12 | 4,56 | 10 | 4,16 | 10 | 3,24 | 7 | 3,72 | 11 | 3,61 | 9 |
| M10 | 2,10 | 13 | 2,22 | 9 | 2,08 | 5 | 1,66 | 5 | 2,04 | 8 | 2,02 | 8 |
| M11 | 5,52 | 48 | 6,77 | 37 | 3,89 | 21 | 4,48 | 19 | 5,71 | 25 | 4,22 | 20 |
| M12 | 3,80 | 16 | 4,68 | 13 | 3,23 | 8 | 3,68 | 8 | 3,00 | 8 | 3,48 | 8 |
| M13 | 2,73 | 11 | 2,87 | 5 | 2,73 | 7 | 2,48 | 5 | 2,33 | 6 | 2,59 | 7 |
| M14 | 2,77 | 17 | 2,65 | 12 | 2,61 | 8 | 1,86 | 7 | 2,45 | 7 | 2,72 | 6 |
| M15 | 6,72 | 32 | 5,52 | 20 | 6,61 | 21 | 6,06 | 18 | 3,85 | 16 | 6,07 | 15 |
| M16 | 3,43 | 9 | 3,42 | 7 | 3,59 | 6 | 2,82 | 6 | 2,87 | 6 | 3,21 | 6 |
| M17 | 4,92 | 13 | 6,77 | 13 | 3,23 | 9 | 3,38 | 10 | 3,86 | 11 | 4,40 | 11 |
| M18 | 2,82 | 16 | 2,87 | 12 | 2,20 | 9 | 2,77 | 4 | 2,18 | 4 | 2,82 | 4 |
| M19 | 2,13 | 7 | 2,21 | 5 | 2,05 | 6 | 1,58 | 3 | 1,90 | 5 | 1,70 | 4 |
| M20 | 2,55 | 41 | 2,74 | 23 | 2,34 | 20 | 2,30 | 20 | 2,16 | 20 | 2,37 | 25 |

# 3. Paternity simulation using 1KG data set

We created a script to analyze the impact of the number of microhaplotypes on the calculation of the paternity probability. We first selected from 1KG data, a male sample and a female sample to be the parents. We then extracted their genotypes for each of the 20 microhaplotypes we want to use. To “create” an artificial child, we selected one haplotype from the male and one from the female as can be seen in Supplementary Figure 1.


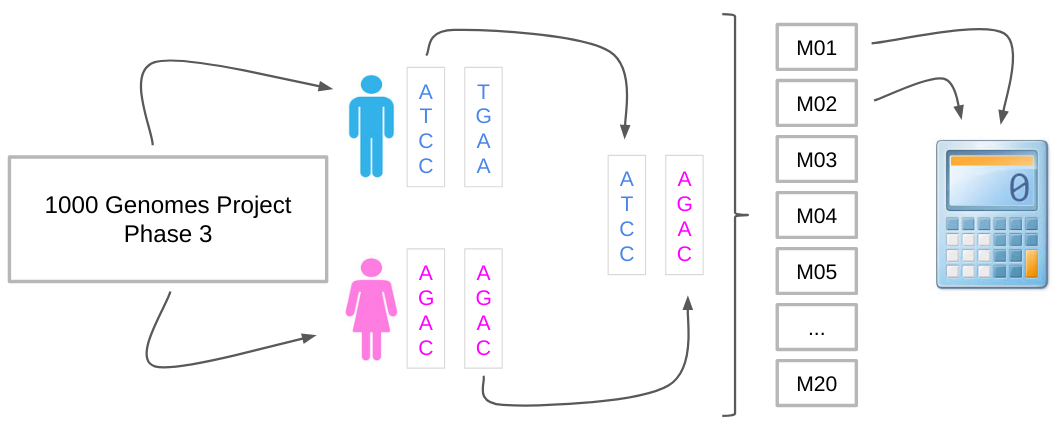


**Supplementary Figure 1:** True paternity simulation using 1KG data. A male sample and a female sample are selected from 1KG data, and their haplotypes are obtained for all 20 microhaplotypes. We “create” a child that inherits one haplotype from the male sample and one from the female sample, for all the microhaplotypes. Finally, we calculate the probability of paternity using an increasing number of microhaplotypes to calculate and the populational frequency.

We used the haplotypes’ population frequencies (considering the 2504 individuals) and calculated the paternity probability, scaling the number of used microhaplotypes in the analysis, from 1 to 20. We called this simulation *Random* and repeated it 26000 times using the whole data. Then, selecting one male and one female from the same population, we repeated the process 1000 times for each population (*Per population*). To do this last procedures, we also used the population frequency of the population from where the samples were selected. We made a boxplot with the result of these simulations, it can be seen in Figure 1 (main article), light grey boxes on *Random* and on *Per population*.

We also decided to test if the opposite situation would be detected by the microhaplotypes. Therefore, we created a script to simulate the false paternity case, a male and a female sample were selected from 1KG. We “created” a child using half of male haplotypes and half of female haplotypes. To test for paternity, we randomly selected a male from 1KG (excluding the male that was used to “create” the child) and calculated the probability of him being the real father, see Supplementary Figure 2. We increased the number of haplotypes and calculated the probability for each number of microhaplotypes being analyzed. This was repeated 26000 times for the *Random* situation and 1000 times for each population for the *Per population* situation (as described above). The result is shown in Figure 1 (main article), and it is represented by the dark grey boxes on *Random* and on *Per population*.


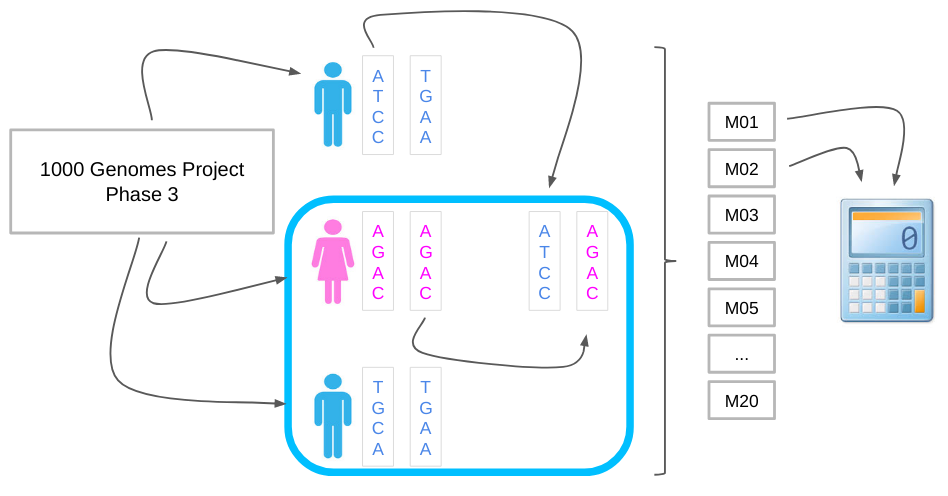


**Supplementary Figure 2:** False paternity simulation using 1KG data. Two male samples and one female sample are selected from 1KG data, and their haplotypes are obtained for all 20 microhaplotypes. We “create” a child that inherits one haplotype from one of the male samples and one from the female sample, for all the microhaplotypes. Finally, we used the second male sample to calculate the probability of paternity using an increasing number of microhaplotypes to calculate and the populational frequency.

# 4. Analysis of mother and alleged father sequencing data

We performed some steps of quality filtering on the sequencing data (Section 2.3 on the main article). The result was a list of haplotypes (alleles) for each microhaplotype of each mother and alleged father. Each list should contain either one or two haplotypes (for each locus with enough quality), since humans are diploid and they can be homozygous or heterozygous. However, for each locus, we encountered more haplotypes than expected. These additional haplotypes can be generated by sequencing errors. To analyze this, we plotted a histogram of all the samples’ haplotypes relative frequencies values (Supplementary Figure 3).


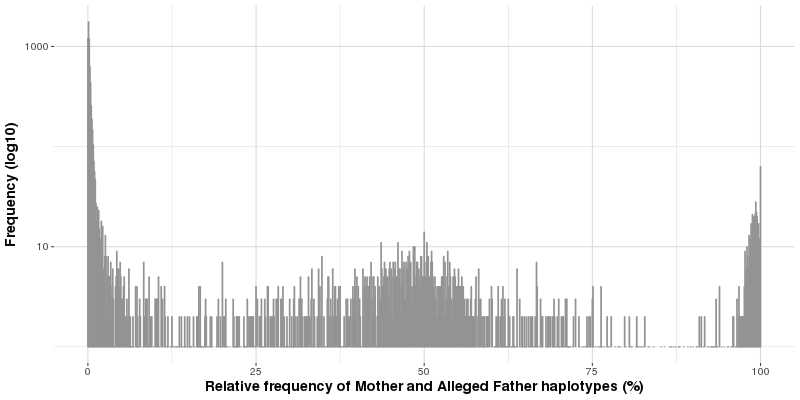


**Supplementary Figure 3:** Histogram of relative frequency of all haplotypes detected in mother and alleged father’s sequencing data.

We can see in Supplementary Figure 3 concentrations around 50% and 100%. These reflect haplotypes at heterozygous and homozygous loci. However, we can see that there is a huge variation in these frequencies, and this is why we have to consider the imbalance inherent in the sequencing method. Based on the review by Nilsen et al [4], a SNP can be considered heterozygous if the allelic imbalance varies between 20% and 80%. In Supplementary Figure 3 we can observe that there is a high concentration of relative frequencies below 20%, and these probably result from sequencing errors.

We analyzed the microhaplotypes’ lists and the relative frequencies of the haplotypes found in the data. In addition to the information obtained from the Supplementary Figure 3, we observed that some haplotypes had an imbalance different from the 20-80% for heterozygous locus. We also noticed that the errors should have relative frequencies below 10%, but some of them were higher than expected.

From the simulations performed with 1KG, we know that analysis of better quality loci will lower the chances of there occurring a false positive (Section 3.1 on the main article). To circumvent errors with high relative frequencies and try and maintain the quality of the data, we adjusted the rules to determine the genotype of the mother and alleged father’s microhaplotypes (Supplementary Table 24).

**Supplementary Table 24:** Sequential criteria enabling the identification of haplotypes in the mother and alleged father, based on the relative frequencies of “haplotypes” observed at a given locus.

| Condition 1 | Condition 2 | Significance |
| --- | --- | --- |
| One “haplotype” >10% | Same “haplotype” >80% | Homozygous |
| Two “haplotypes” >10% | One “haplotype” >80% | Homozygous |
| Two “haplotypes” >10% | Two “haplotypes 20%-80% | Heterozygous |
| Three “haplotypes” >10% | Two “haplotypes” >35% | Heterozygous |

# 5. Analysis of plasma sample and fetal frequency

According to Saba et al [5], the appropriate foetal fraction interval to correctly identify the paternal haplotype is between 1.4% and 11%. To verify that the observed foetal fractions were inside this interval, we examined trios in which the alleged father was known to be the true father. We performed the quality filtering steps (as performed in mother and alleged father’s sequencing data) and obtained a list of haplotypes. Then we selected all haplotypes that were different from the mother but that matched the alleged father. We made a histogram of the relative frequencies of all these haplotypes (from all the trios), Supplementary Figure 4.

**
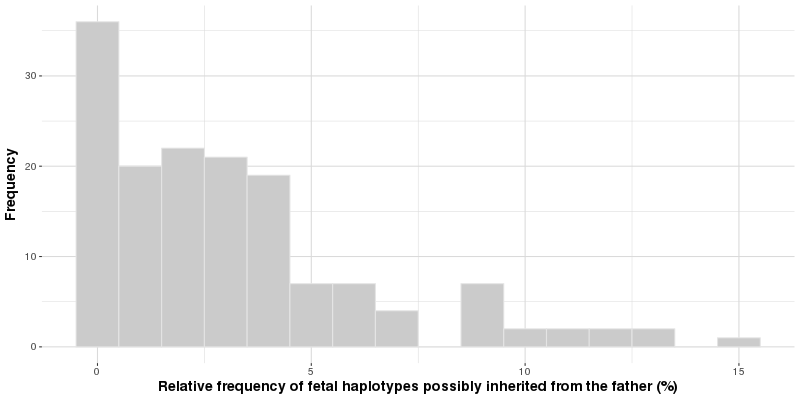
**

**Supplementary Figure 4:** Histogram of relative frequency of haplotypes possibly inherited only from the father. It is expected that the relative frequencies should be below 11%.

We can observe that most of them are between 1.4% and 11%, but there are some exceptions. A foetal fraction derived solely from the alleged father and greater than 12.5% would imply a total foetal fraction of approximately 25%, which could be discarded given the known gestational ages of the participating mothers. We observed that the errors inherent to the sequencing technique had relative frequencies in the same range as that of the foetal paternal haplotypes. Conversely, very low foetal fractions could not be accepted. Therefore, taking these observations into account and seeking to obtain reliable information, we established that the foetal fraction detection interval should be between 1% and 12%.

# **6. Evidence for paternity**

To analyze the sequence data and conclude that the alleged father is the true father, we formulated the evidence for paternity. Given a microhaplotype, *m*, we obtained the number of haplotypes shared between each mother (*M*) and alleged father (*SP*), |*SP_m_*∩*M_m_*|. We obtained the number of haplotypes shared between the plasma (relative frequencies in the interval of 1% and 12%, and different from the mother’s haplotypes) and the alleged father, |*FF_m_*|. For each combination of |*SP_m_*∩*M_m_*| and |*FF_m_*|, we have a different value for the evidence for paternity (*EV_m_*) and the proposed result.

**Supplementary Table 25:** Combinations of haplotypes in alleged father, mother and plasma, the value given for evidence of paternity and the result. Given a microhaplotype *m*, we analyze the observed haplotypes and determine whether or not there is evidence that the alleged father is the true father. |*SP_m_* ∩ *M_m_*| is the number of haplotypes shared between alleged father and mother. |*FF_m_*| is the number of plasma haplotypes that matched the alleged father’s haplotypes and are different from the mother’s (they are within the interval of 1% and 12%). *EV_m_* is the evidence for paternity and the result is if the given combination can support the paternity or not.

| **\|*SP_m_*∩*M_m_*\|** | **\|*FF_m_*\|** | ***EV_m_*** | **Result** | **\|*SP_m_*∩*M_m_*\|** | **\|*FF_m_*\|** | ***EV_m_*** | **Result** |
| --- | --- | --- | --- | --- | --- | --- | --- |
| 0 | 0 | 0 | Cannot be father | 1 | 0 | 0.5 | Can be father |
| 0 | 1 | 1 | Can be father | 1 | 1 | 1 | Can be father |
| 0 | 2 | 1 | Can be father | 2 | 0 | 0.5 | Can be father |

We obtained the total number of microhaplotypes with quality to be analyzed for each trio. Each microhaplotype was classified using the combinations given in Supplementary Table 25. The result is presented in Supplementary Table 26, and we also supply a label for each trio (obtained with another method), if it is an inclusion of paternity, the label is “I”, and if it is an exclusion, the label is “E”.

**Supplementary Table 26:** List of analyzed trios. List of trios used in this analysis, their true result, if inclusion = “I” and if exclusion = “E”. The total number of microhaplotypes that passed the quality filtering. The number of microhaplotypes with evidence for paternity: equal 1 *EV*(1); equal 0.5 *EV*(0.5); and equal 0 *EV*(0). The calculated value for *W*.

| **Trio** | **Label** | **Total** | ***EV*(1)** | ***EV*(0.5)** | ***EV*(0)** | ***W*** |
| --- | --- | --- | --- | --- | --- | --- |
| T01 | E | 17 | 4 | 10 | 3 | 9.84840e-11 |
| T02 | I | 15 | 6 | 8 | 1 | 0.93616 |
| T03 | E | 15 | 4 | 10 | 1 | 0.15259 |
| T04 | I | 12 | 3 | 9 | 0 | 0.99566 |
| T05 | I | 12 | 3 | 8 | 1 | 0.40075 |
| T06 | I | 14 | 6 | 7 | 1 | 0.07337 |
| T07 | I | 14 | 5 | 9 | 0 | 0.99958 |
| T08 | I | 14 | 7 | 7 | 0 | 0.99999 |
| T09 | I | 14 | 5 | 9 | 0 | 0.99994 |
| T10 | E | 15 | 6 | 5 | 4 | 3.58403e-15 |
| T11 | E | 16 | 2 | 13 | 1 | 0.05027 |
| T12 | I | 15 | 4 | 10 | 1 | 0.10703 |
| T13 | I | 15 | 7 | 7 | 1 | 0.99635 |
| T14 | E | 18 | 2 | 12 | 4 | 1.81796e-16 |
| T15 | E | 17 | 2 | 13 | 2 | 2.72760e-06 |
| T16 | E | 12 | 1 | 9 | 2 | 1.05139e-07 |
| T17 | E | 14 | 1 | 11 | 2 | 2.78312e-07 |
| T18 | I | 13 | 7 | 5 | 1 | 0.14035 |
| T19 | E | 12 | 0 | 9 | 3 | 3.01697e-12 |
| T20 | E | 14 | 0 | 12 | 2 | 4.16158e-07 |
| T21 | I | 14 | 2 | 12 | 0 | 0.99078 |
| T22 | E | 15 | 4 | 7 | 4 | 1.35272e-14 |
| T23 | E | 15 | 2 | 11 | 2 | 4.82318e-06 |
| T24 | I | 17 | 6 | 11 | 0 | 0.99998 |
| T25 | E | 16 | 3 | 9 | 4 | 1.50207e-15 |
| T26 | I | 16 | 6 | 10 | 0 | 0.99999 |
| T27 | I | 14 | 5 | 8 | 1 | 0.45376 |
| T28 | I | 15 | 7 | 8 | 0 | 0.99996 |
| T29 | I | 17 | 7 | 10 | 0 | 0.99997 |
| T30 | I | 14 | 9 | 5 | 0 | 0.99991 |
| T31 | E | 15 | 3 | 9 | 3 | 5.19178e-12 |

We expect that in cases of inclusion, the number of *EV*(0) is equal to zero. However, we observed that, for the cases of inclusion, seven trios had one microhaplotype indicating the exclusion of paternity (T02, T05, T06, T12, T13, T18 and T27). In the cases of exclusion, only two of them had only one *locus* indicating that the alleged father cannot be the father (*EV_m_* = 0), the others had two or more exclusionary microhaplotypes.

To investigate further these discrepancies, we evaluated the information relating to the discordant microhaplotype. This is presented in Supplementary Table 27.

**Supplementary Table 27:** List of trios that have discrepant results.

| **Trio** | **Locus** | **Problem** |
| --- | --- | --- |
| T02 | M04 | There is no haplotype in common between the alleged father and plasma. |
| T05 | M04 | The haplotype observed in the alleged father has a relative frequency of 0.47%. |
| T06 | M08 | There is no haplotype in common between the alleged father and plasma. |
| T12 | M04 | The haplotypes observed in the alleged father have relative frequencies of 0.98% and 0.07%. |
| T13 | M11 | The haplotype observed in the alleged father has a relative frequency of 0.74%. |
| T18 | M05 | The haplotypes observed in the alleged father have relative frequencies of 0.40% and 0.20%. |
| T27 | M03 | The haplotype observed in the alleged father has a relative frequency of 0.01%. |

We can see in Supplementary Table 27 that the false exclusion of paternity results from not detecting the paternal haplotype. Five cases occurred because the relative frequency was below the minimum (1%) of detection. The two remaining cases (T02 and T06) were due to the father’s haplotype not being present in the sequencing data. By analyzing the cases in which there was only one exclusionary locus (*EV*(0) = 1), we can see that seven of them are false negatives and two are true negatives. Following traditional paternity testing, we stipulated that only one exclusionary microhaplotype is insufficient evidence and that at least two discrepant loci are required to conclude in the exclusion of paternity. Merely one discrepant locus was considered to be inconclusive.

# 7 References

1. Debeljak M, Freed DN, Welch JA et al. Haplotype counting by next generation sequencing for ultrasensitive human DNA detection. Mol Diagn. 2014;16(5):495–503.
2. Auton A, Abecasis G, Altshuler D et al. A global reference for human genetic variation. Nature. 2015; 526:68–74.
3. Rychlik W. OLIGO 7 Primer Analysis Software. Methods Mol Biol. 2007;402:35–59.
4. Nielsen R, Paul JS, Albrechtsen A, Song YS. Genotype and SNP calling from next-generation sequencing data. Nat Rev Genet. 2011;12(6):443–451.
5. Saba L, Masala M, Capponi V, Marceddu G, Massidda M, Rosatelli MC. Non-invasive prenatal diagnosis of beta-thalassemia by semiconductor sequencing: a feasibility study in the sardinian population. Eur J Hum Genet. 2017;25(5):600–607.
